# Microstructural Changes Due to Alkali-Silica Reaction during Standard Mortar Test

**DOI:** 10.3390/ma8125450

**Published:** 2015-12-02

**Authors:** Jun-Ho Shin, Leslie J. Struble, R. James Kirkpatrick

**Affiliations:** 1 Department of Civil and Environmental Engineering, University of Illinois, Urbana, IL 61801, USA; lstruble@illinois.edu; 2 Department of Geology, University of Illinois, Urbana, IL 61801, USA

**Keywords:** alkali-silica reaction, backscattered electron imaging, silica glass, ASTM C1260, microcracking, microstructure

## Abstract

The microstructural development of mortar bars with silica glass aggregate undergoing alkali-silica reaction (ASR) under the conditions of American Society for Testing and Materials (ASTM) Standard Test C1260 was analyzed using scanning electron microscopy and qualitative X-ray microanalysis. Cracking in the aggregate, the hydrated paste, and the paste-aggregate interface was important in the development of the microstructure. Cracks were characterized according to their location, their relationship to other cracks, and whether they are filled with ASR gel. Expansion of the bars was approximately 1% at 12 days and 2% at 53 days. They fell apart by 63 days. The bars contained two zones, an inner region that was undergoing ASR and an outer and much more highly damaged zone that extended further inward over time. Evidence of ASR was present even during the period when specimens were immersed in water, prior to immersion in NaOH solution.

## 1. Introduction

Alkali-silica reaction (ASR) occurs in concrete by reaction of alkali metal ions and hydroxyl ions that are derived mainly from the cement and certain forms of silica that may be present in the aggregate. The product of the reaction is a hydrated ASR gel that often also contains calcium. The gel is able to imbibe water and swell, leading to internal swelling pressure, expansion, cracking, and loss of strength in concrete structures. For ASR to take place in concrete, it is generally acknowledged that three conditions are necessary [1]:

1. a sufficient quantity of reactive silica, usually derived from the aggregate,
2. sufficient alkali (Na and/or K), usually derived from the cement paste, and
3. sufficient moisture, thought to be not less than 80% relative humidity in the concrete pores.

If any of the above conditions is lacking, the expansion will not occur.

Although ASR has been the focus of worldwide attention since it was first observed and described, and the available literature on it is extensive, the molecular and microstructural mechanisms by which ASR proceeds are quite complex and remains poorly understood. The reaction is initiated by the attack of hydroxyl ion and the dissolution of silica present in the aggregate. Subsequently, the first reaction products become ASR gel. Only in a later stage, when ASR has already fully developed, the ASR products extrude the aggregates, take-up calcium and come close to the composition of C–S–H [2,3,4]. Hou and colleagues [5,6,7,8] in our laboratory described the following sequence for ASR gel formation based on laboratory studies of mortar specimens. Silica dissolved due to the attack by hydroxyl ions reacts with portlandite (CH) to produce depolymerized, Ca-rich calcium-silicate-hydrate (C–S–H) until the neighboring CH is locally consumed. At this point, the C–S–H becomes richer in silica and more polymerized. Only then does a hydrous ASR gel start to form. This conclusion is consistent with the suggestion of Taylor [9] that the chemistry of ASR is essentially that of a pozzolanic reaction. More recently, Li, *et al.* [7] confirmed these reaction steps and showed that consumption of CH and reaction of C–S–H in the paste prior to formation of ASR gel can be greater at 80 °C than at ambient temperature. This study, and the study of Hou, *et al.* [8], were done at 80 °C, and the widely used American Society for Testing and Materials (ASTM) standard test C1260 specifies this temperature. This reaction sequence is the starting point for the microstructural interpretations presented here.

The overall project is described in more detail in the PhD thesis of the first author [10]. The objective of the entire project is to understand the mechanical behavior of concrete during ASR based on the microstructural changes caused by the reaction. This paper presents the microstructural analysis, which provides the basis for the subsequent mechanical modeling.

There are several standard tests for identifying alkali-reactive aggregates currently in use, and ASTM has four such tests for mortar or concrete. Of these, the Standard Test Method for Potential Alkali Reactivity of Aggregates (ASTM C1260, the accelerated mortar bar test) was used in the work reported here. This test was developed in South Africa at the National Building Research Institute [11,12,13] and is known to be effective in identifying reactive aggregates [14].

## 2. Experimental Section

Three mortar bars were prepared in accordance with the ASTM C1260 test procedure, one for microstructural examination and two for measuring expansion. Table 1 summarizes the mix proportions and environmental conditions. The mortars contained silica glass (Vycor 7913, Corning Inc., Corning, NY, USA) as the only aggregate and a portland cement (ASTM Type I, Lone Star Industries Inc., Indianapolis, IN, USA) with a total alkali content of 0.8%. The chemical composition of Vycor used is shown in Table 2 [15]. The silica glass obtained from the manufacturer was sieved to meet the fine aggregate grading requirements in Table 3. Because the two largest sizes of aggregate prescribed by C1260 were unavailable, their amounts were included in the third size. The batch was mixed using an electrically driven paddle mixer (N-50, Hobart Corp., Troy, OH, USA) as described in ASTM C305-99, Standard Practice for Mechanical Mixing of Hydraulic Cement Pastes and Mortars of Plastic Consistency. The mortar bars were cast in 25 mm × 25 mm steel molds having a 285-mm length, as specified in ASTM C490-00, Standard Practice for Use of Apparatus for the Determination of Length Change of Hardened Cement Paste, Mortar, and Concrete. To provide uniform compaction in all regions of the molds, two mortar layers of equal thickness were compacted with a rubber tamper. The bars were stored in the molds in a moist room for 24 h and then demolded and stored in water at a temperature of 80 °C for another 24 h. The initial length of each bar was measured, and then all the bars were immersed in 1-N NaOH solution and stored at 80 °C for the remainder of the test. Two bars were removed from solution periodically and their lengths measured. This measurement was repeated periodically until 56 days, well beyond the 14-day duration of the standard test.

Length measurements were made using a length comparator, as described in ASTM C490. Each specimen length was the difference between the value measured for the specimen and the value measured for the reference bar. The strain at each age, expressed as a percent, was the difference between the initial length and the length at that age. Throughout this study, zero time refers to the time when all bars are first immersed in 1-N NaOH, so the mortar was prepared at −2 days and the bars were demolded at −1 day.

**Table 1:** General description of specimens used for this study.

**Aggregate Size**	1.18–0.15 mm
**Aggregate-cement ratio**	2.25
**w/c**	0.47
**Specimen size**	25 mm × 25 mm × 285 mm
**Alkali content of cement**	0.8
**Environment**	moist room at 23 °C for 24 h + immersed in 80 °C water for 24 h + immersed in 80 °C NaOH solution until specimen preparation

**Table 2:** Chemical composition of Vycor 7913 used for this study [15].

Chemical Composition	(% Approx)
SiO_2_	96.4%
B_2_O_3_	3.0%
Al_2_O_3_	0.5%
Miscellaneous Traces	0.1%

**Table 3:** Grading of aggregate specified in ASTM C1260 and used in the study.

Sieve Size		Mass Specified in C1260		Used Mass	
Passing	Retained on	%	Gram	%	Gram
4.75 mm (No. 4)	2.36 mm (No. 8)	10	99	0	0
2.36 mm (No. 8)	1.18 mm (No. 16)	25	247.5	0	0
1.18 mm (No. 16)	0.6 mm (No. 30)	25	247.5	60	594
0.6 mm (No. 30)	0.3 mm (No. 50)	25	247.5	25	247.5
0.3 mm (No. 50)	0.15 mm (No. 100)	15	148.5	15	148.5

Polished specimens for SEM examination were prepared from the third mortar bar at various ages. Cross sectional specimens were cut from the bar using a small saw (Isomet 2000 Precision Saw, Buehler, Inc., Lake Bluff, IL, USA) equipped with a thin diamond blade and operating at a low speed (400 rpm). Propylene glycol was used as a lubricant and was removed by ultrasonic treatment in ethanol. Specimens were dried at 60 °C for 5 days (a temperature selected to limit shrinkage cracking [16,17,18]). They were then impregnated with a very low viscosity epoxy (EpoxySet, Allied High Tech Products, Inc., Rancho Dominguez, CA, USA) under vacuum and cured at atmospheric pressure at room temperature (23 °C) for 10 h. The specimens were then ground and polished using propylene glycol as the lubricant. The grinding sequence utilized grinding discs impregnated with silicon carbide—first a coarse size (120 grit (116 µm)) to remove major imperfections and excess epoxy, followed by finer sizes to produce a smooth and flat surface (180 grit (78 µm), 240 grit (66 µm), 320 grit (40 µm), 400 grit (34 µm), 600 grit (28 µm) and 800 grit (22 µm)). The polishing sequence utilized a lapping wheel, a low-relief polishing cloth, and 1-µm diamond paste followed by ¼-µm diamond paste. After each grinding and polishing step, the surface condition was examined using an optical microscope and the specimen was ultrasonically cleaned in ethanol. The polished specimens were then carbon-coated for 3–5 s for SEM analysis using a Polaron SC7620 Sputter Coater (Quorum Technologies, East Grinstead, West Sussex, UK).

The SEM examination utilized a microscope (JEOL JSM-6060LV Low Vacuum, Tokyo, Japan) equipped with a quadrupole backscattered electron (BSE) detector and an energy-dispersive X-ray (EDX) detector. The accelerating voltage was 20–30 kV. For systematic investigation of the entire cross section of the specimens, BSE micrographs (so-called montages) were created by combining 12, 16, or 20 individual images obtained at low magnifications (11–14×). BSE micrographs were created in selected areas at higher magnifications. EDX spectra were collected for selected microstructural features.

## 3. Results and Discussion

**Figure 1:**
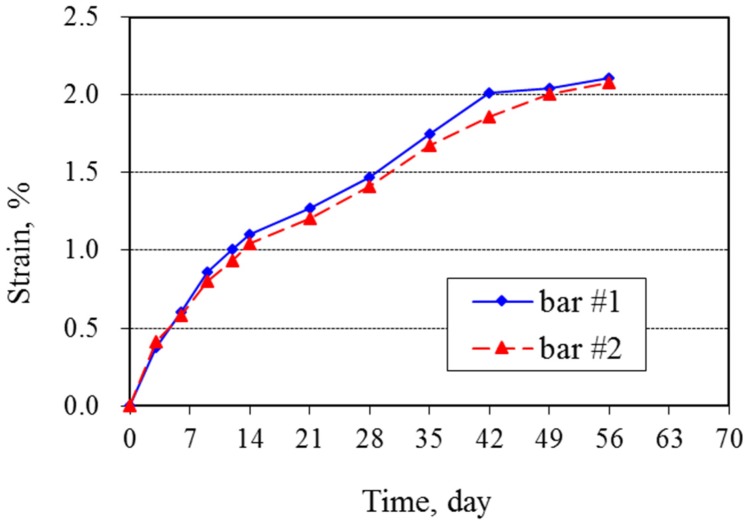
Expansion of mortar bars tested under ASTM C1260.

**Figure 2:**
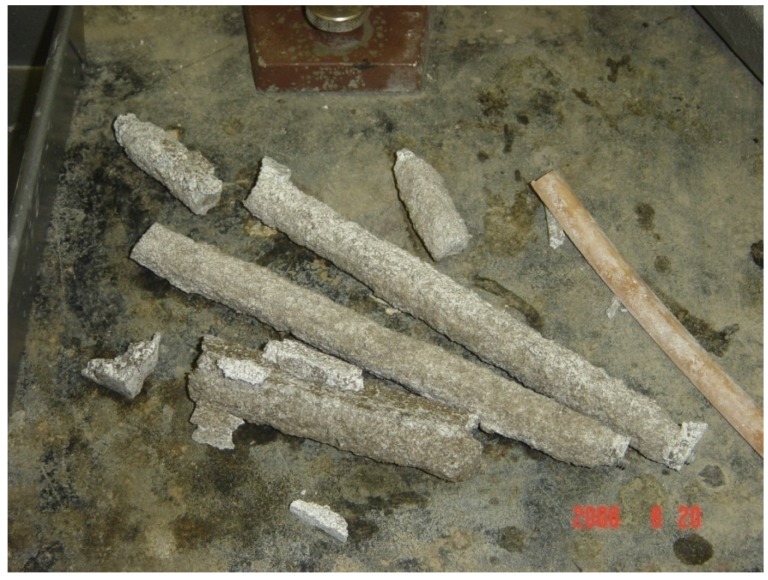
Mortar bars severely deteriorated after immersion in NaOH solution for 63 days.

The expansion measurements show strains of about 1% at 12 days and 2% at 56 days (Figure 1). The rate of expansion decreased progressively with increasing time. Bars were still expanding at 63 days, when the test was terminated because they had so severely deteriorated that no further measurements could be made (Figure 2). The duplicate bars showed only slight differences in expansion, differing by only 1% to 4% of the mean expansion. The ASTM C1260 criterion for a potentially deleterious aggregate is expansion greater than 0.2% at 14 days, and the results clearly show, as expected, that this silica glass is highly reactive.

**Figure 3:**
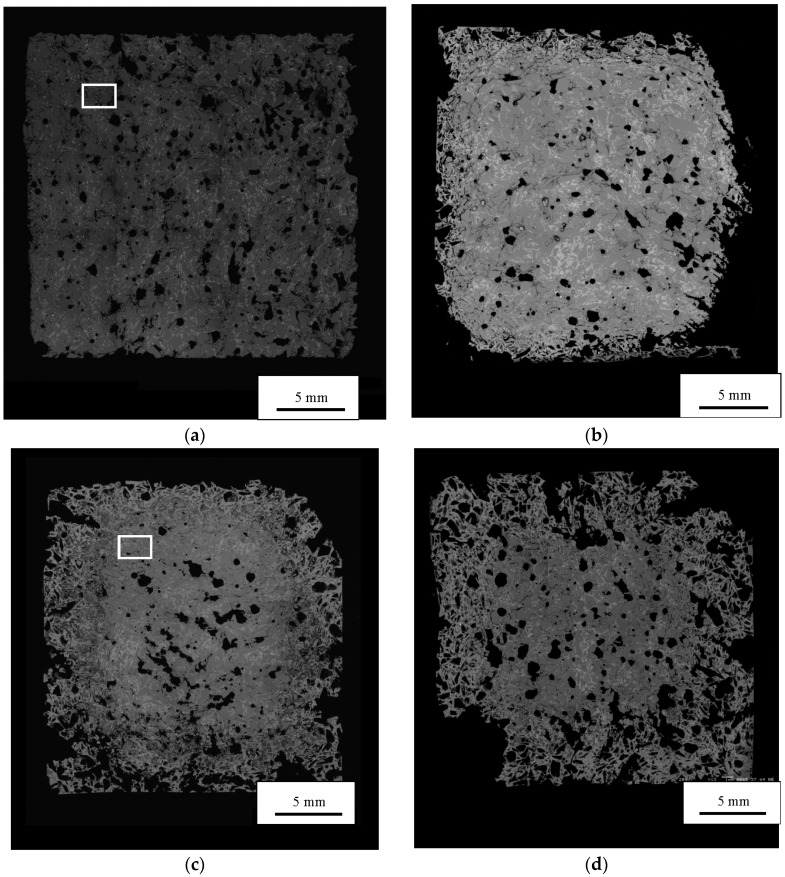
BSE images of entire cross sections of mortar bars at 0 (**a**); 3 (**b**); 21 (**c**); and 42 (**d**) days.

The SEM montages at 0, 3, 21, and 42 days show relatively unreacted cores surrounded by progressively thicker regions that are much more damaged. The outer regions contain numerous voids (Figure 3). Many of these voids are angular in shape and appear to be spaces left when aggregate particles were dissolved. Although the specimens were handled and prepared very carefully, they became very fragile and the montages show that their edges were considerably damaged.

In the cores, higher magnification SEM images show extensive cracking. The cracks are approximately 3–20 µm in width. The number and width of the cracks increase progressively with increasing time. As discussed below, these cracks are attributed to ASR.

**Figure 4:**
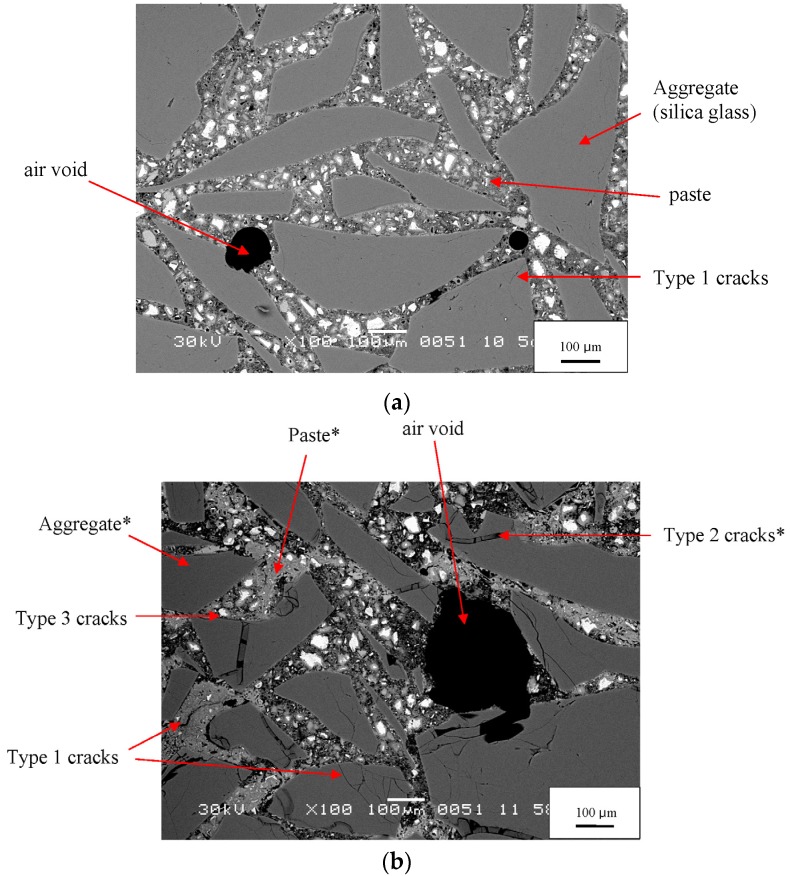
BSE images of a mortar at 0 days (**a**) and at 21 days (**b**). The white boxes in Figure 3a,c show where in the montage these two images were taken. Labels are explained in the text. Note: * denotes constituents whose EDX data are provided in Table 4.

The SEM images of the 0-day sample illustrate key microstructural features—aggregate particles, paste, air voids, and microcracks (Figure 4a). Constituents are easily identified. White areas are unhydrated cement grains, light gray areas are hydrated cement phases, dark gray areas are aggregate particles, and black areas are epoxy-filled pores. By 21 days, ASR has clearly begun, its reaction products are clearly visible, and the microstructure has become more complicated (Figure 4b). The number of microcracks in the aggregate grains has greatly increased, and many are gel-filled, as previously observed by Hou, *et al.* [8] and Li, *et al.* [7]. Unhydrated cement is still present, but its amount is much less than at 0 days. An inhomogeneous spatial distribution of porosity is apparent in the paste, a feature reported by Diamond [19,20]. Two types of paste can be seen: bright, dense, almost nonporous regions with fewer unhydrated cement grains and relatively dark, highly porous regions with more numerous unhydrated cement grains.

The EDX data collected for specific microstructural features in Figure 4b provide additional support for identifying these features throughout the study (Table 4). The aggregate (silica glass) shows peaks for only silicon and oxygen. The hydrated paste shows a strong peak for calcium along with lesser peaks for silicon and oxygen and other elements. The ASR gel shows a strong peak for silicon and lesser peaks for sodium, calcium, and oxygen. The very small peaks for chlorine observed in some spectra are presumed to originate from the epoxy used to impregnate the specimens. Throughout this study, EDX spectra with strong peaks for silicon and small peaks for sodium or potassium and perhaps with small peaks for calcium are presumed to indicate ASR gel.

**Table 4:** EDX chemical analysis of aggregate, paste, and gel shown in Figure 4b.

Element	Na	Si	K	Ca	Mg
Aggregate	–	3.5	–	–	–
Paste	–	0.8	0.05	3.1	0.05
Gel	0.2	3.4	–	0.4	–

Based on analysis of hundreds of SEM images and X-ray microanalyses, we have classified microcracks in the reacted specimens as follows:

1. Type 1—cracks without gel isolated from each other;
2. Type 2—cracks fully or partially filled with gel;
3. Type 3—cracks without gel but connected to a Type 2 crack.

Table 5 summarizes the types of cracks and their microstructural locations at various ages.

**Figure 5:**
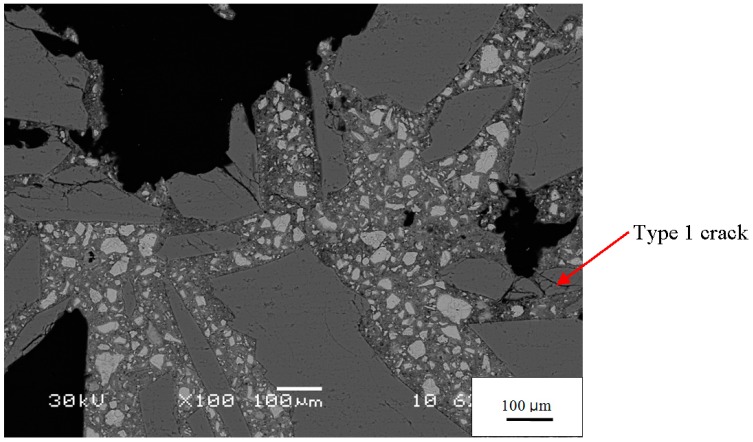
BSE image of the −1 day mortar showing the irregular, angular shape of the aggregate grains and their unfilled cracks.

An example of Type 1 cracks, with no gel and isolated from each other, is seen in the 21-day mortar in Figure 4b. These are empty cracks in aggregate particles. Other empty cracks are observed in the paste and at the paste-aggregate interface. Although the specimen has developed considerable expansion by this age (1.24%) and the figure exhibits other microstructural features associated with ASR, the isolation of these cracks suggests they are not directly related to ASR. Even before ASR started, some aggregate particles were fractured, as shown in Figure 5, probably because, as described above, the aggregate was crushed, ground, and sieved to meet the grading requirement for mortar bars. Therefore the Type 1 cracks do not appear to be directly associated with ASR and were likely present prior to testing. Nonetheless, they probably play an important role in ASR expansion by concentrating stresses, enhancing crack growth, and serving as a locus of hydroxide attack on the aggregate.

**Table 5:** Summary of crack types and locations at various ages.

Time		−1 Day	0 Day	3 Days	21 Days	42 Days
In the aggregates	Type 1	O	O	O	O	O
	Type 2	X	O	O	O	O
	Type 3	X	X	X	X	X
At aggregate–paste interface	Type 1	O	O	O	O	O
	Type 2	X	X	X	X	X
	Type 3	X	X	O	O	O
In paste	Type 1	O	O	O	O	O
	Type 2	X	X	X	X	X
	Type 3	X	X	O	O	O

Notes: Type 1 cracks have no gel and are isolated from each other; Type 2 cracks are fully or partially filled with gel; Type 3 cracks have no gel and are connected with a Type 2 crack. O: observed, X: not observed.

The Type 2 cracks, fully or partially filled with gel, were observed only in aggregate grains. An example is shown in Figure 4b. Because they contain gel, they are clearly associated with ASR. The gel is itself extensively cracked, probably due to shrinkage during drying. This microstructure is essentially identical to the gel-filled cracks observed in mortars with silica glass aggregate by Hou, *et al.* [8] and by Li, *et al.* [7]. The gel is slightly darker than the surrounding silica glass, indicating that its average atomic number is slightly lower than that of the silica. Type 2 cracks were seen to increase in number and width with increasing reaction time. In our samples gel never occurred outside the aggregate grains. In contrast, previous studies with more rapidly reactive aggregates, such as Beltane opal, have shown that samples produce much larger amounts of gel that can be seen in cracks through the cement paste, along the aggregate-paste interface, and in air voids [9,21,22,23].

The Type 3 cracks, which contain no gel but are connected to gel-filled cracks, are observed in the paste and at the paste-aggregate interface. An example is shown in Figure 4b. Because they are connected to cracks containing gel, they appear to be associated with ASR. The gel can imbibe moisture and exert significant pressure on the surrounding mortar. Hobbs [24], for instance, suggested that the stresses induced by gel swelling causes cracking at neighboring paste-aggregate interfaces and in the paste. These cracks are likely to initiate and grow where the stresses are highest, probably near gel, and it is expected that the Type 3 cracks would form where Type 2 cracks terminate at the exterior surfaces of aggregate particles. Some Type 1 cracks could actually be Type 3 cracks for which the relationship to Type 2 cracks was not observed due to the two-dimensional surfaces of SEM analysis. Type 3 cracks and perhaps some of the Type 1 cracks probably developed due to tensile stresses in the mortar bar caused by gel expansion that exceeds the tensile strength of the concrete. These cracks only occur in the paste or at the aggregate-paste interface because these regions are generally weaker than aggregate particles, especially the aggregate-paste interface, which is often cracked due to shrinkage.

**Figure 6:**
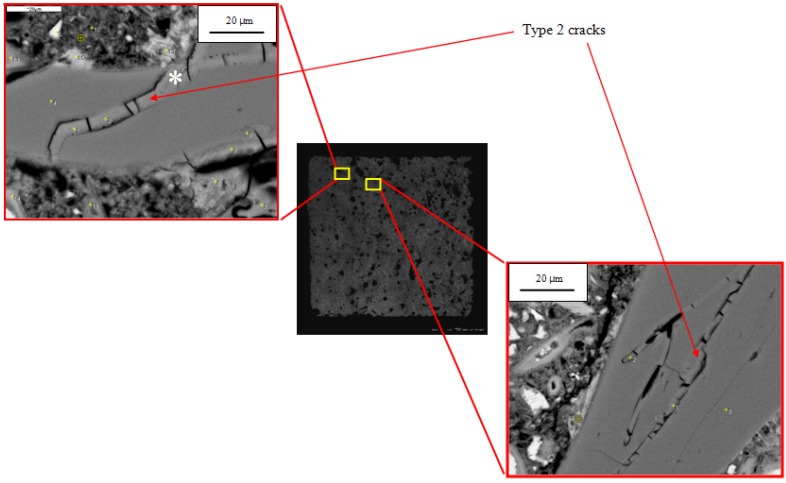
BSE images of ASR gel in aggregate grains in the 0-day specimen.

Although no gel was observed at −1 days, some had already formed at 0 days. The appearance and composition of the gel, shown in Figure 6, are clearly associated with ASR. The gel composition in Table 6 is different from that formed later, such as the gel shown in Table 4. The earlier gel is higher in potassium and lower in sodium, presumably reflecting the portland cement as the only source of alkali at this time. Thus, ASR began while the bar was immersed in hot water and before it was immersed in NaOH solution. This was only observed in the one micrograph shown, but nonetheless it was an interesting and unexpected finding.

**Table 6:** EDX chemical analysis of gel in Figure 6 marked with *.

Element	Na	Si	K	Ca	Mg
gel	–	1.0	0.2	0.2	–

Although the microstructural development due to ASR in the central core regions of the specimens is quite similar to that previously observed by Hou, *et al.* [8] and Li, *et al.* [7] for mortars with silica glass aggregate subjected to procedures similar to ASTM C1260, the highly altered exterior rims have not been previously described. As shown in the montage images (Figure 3), these rims contain numerous voids and do not retain the cracks associated with ASR discussed above. Many of the voids appear to be partially or fully dissolved silica glass fragments.

**Figure 7:**
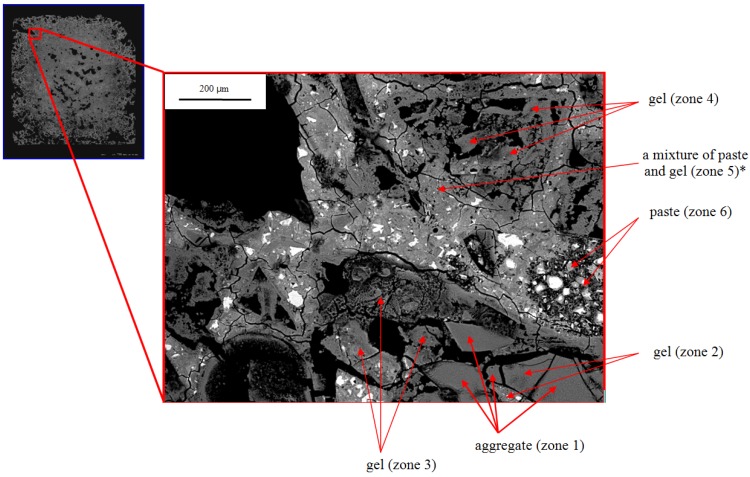
BSE image from 21-day-old mortar in the top left section of specimen.

The SEM images of the rim of the 21-day sample provide more detail. In Figure 7, the lower center and right of the micrograph (zones 1–3) is a partially altered grain of silica glass. EDX analysis shows that only zone 1 is unaltered glass and the others are ASR gel. Similarly, the triangular shaped region in the upper right (zone 4) appears to be a silica glass particle that has fully reacted to form gel. Other regions contain extensively cracked cement paste (e.g., zones 5, 6). EDX shows that some points in the paste contain considerable sodium (e.g., zone 5), much more than observed in the paste in the core and more calcium than observed in the gel in the core (Table 4 and Table 7); and these points are therefore labeled as a mixture of paste and gel. One major difference between the exterior and interior zones is that in the exterior the aggregate has been much more extensively attacked and ASR gel development is much greater.

**Table 7:** EDX chemical analysis of zone 5 marked with * in Figure 7.

Element	Na	Si	K	Ca	Mg
Zone 5	0.1	0.9	–	0.8	–

**Figure 8:**
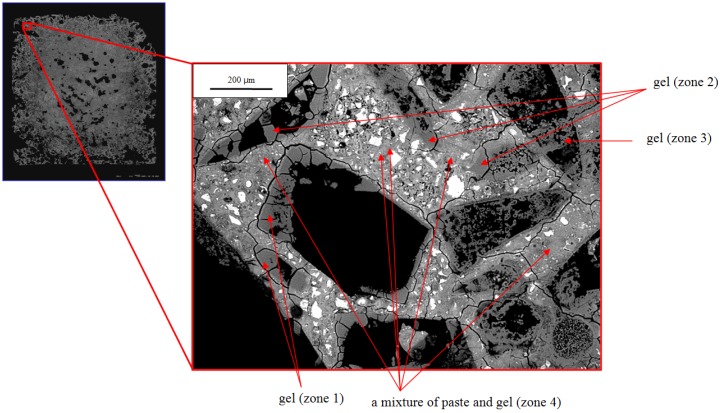
BSE image from 21-day-old mortar at different location.

The microstructure closer to the exterior surface of the sample shows even more extensive alteration than the interior part of the rim (Figure 8). All regions that appear morphologically to have been silica glass are either fully dissolved or partially replaced by ASR gel, sometimes as a rim around the exterior of the hole. Many of these gel regions are fractured (zone 1 in Figure 8) or highly porous (zone 3 in Figure 8). All points in the paste (except unreacted cement) contain a considerable amount of sodium and are therefore presumed to be a mixture of paste and gel. Based on spectroscopic analyses of Hou, *et al.* [8], it seems likely that the C-S-H is silica-rich and highly polymerized. The outer rims of the bars developed so many voids that they could not sustain their own weight and broke into pieces by 63 days (Figure 2). The presence of the delicate structures in Figure 7 and Figure 8 indicate that the gel was not significantly abraded or removed during grinding and polishing, although some plucking was certainly possible. Thus, we presume that the voids occur because aggregate dissolves during reaction and is not fully replaced by gel.

Mechanistically, these results show that, for this highly reactive aggregate, ASR starts nearly immediately, prior to immersion of the mortar bars in NaOH solution. They also show that ASR proceeds from the exterior to the interior of the bars, presumably as the NaOH diffuses inward. In addition to cracking, continuing reaction causes extensive dissolution of the aggregate and substantial formation of ASR gel. These changes were sufficient to cause the bars to fall apart by 63 days.

In the present study, a mortar test was accelerated by a high temperature of 80 °C and additional NaOH with highly reactive SiO_2_ to assess the mechanical behavior of concrete during ASR. The combination of highly reactive aggregates, high temperature and high alkalinity results in the formation of a microstructure that shows little similarities to the microstructure of ASR-suffering structures. Consequently, the results obtained in these accelerated tests are restricted to the chosen materials and test conditions and are unlikely to be transferrable for real concrete structures.

## 4. Conclusions

Mortar bars containing silica glass aggregate tested using the accelerated mortar bar test, ASTM C1260, rapidly developed expansion due to ASR. Microstructural and compositional analysis shows that the mortars developed a complex structure consisting of an inner region that was undergoing ASR and a fragile, porous outer rim that was more strongly reacted.

Three types of cracks were observed in the inner region: Type 1 cracks in the paste, aggregate grains and at the paste aggregate interface, isolated from each other and other cracks, and containing no gel; Type 2 cracks only in aggregate grains and fully or partially filled with gel; and Type 3 cracks in the paste or at the aggregate-paste interface, containing no gel and always connected to a Type 2 crack.

The outer rims of the samples were much more severely damaged than the cores due to alkali attack and damage progressed into the bars with increasing time.

ASR began when the bar was immersed in water, prior to immersion in NaOH solution.
